# The future of financing for HIV services in Uganda and the wider sub-Saharan Africa region: should we ask patients to contribute to the cost of their care?

**DOI:** 10.1186/s12889-016-3573-0

**Published:** 2016-08-27

**Authors:** Tom Kakaire, Walter Schlech, Alex Coutinho, Richard Brough, Rosalind Parkes-Ratanshi

**Affiliations:** 1 Infectious Diseases Institute, Makerere University College of Health Sciences, PO Box 22418, Kampala, Uganda; 2 Dalhousie University Faculty of Medicine, Nova Scotia, Canada

**Keywords:** Financing, Sustaining, Uganda, HIV, Treatment

## Abstract

Whilst multi-lateral funding for HIV/AIDS dramatically increased from 2004 to 2008, it has largely plateaued in the last 8 years. Across sub-Saharan Africa, up to 20 % of total spending on health is used for HIV services, and of this over 85 % is estimated to come from international funding rather than in-country sources. In Uganda, the fiscal liability to maintain services for all those who are currently receiving it is estimated to be as much as 3 % of Gross Domestic Product (GDP). In order to meet the growing need of increased patient numbers and further ART coverage the projected costs of comprehensive HIV care and treatment services will increase substantially. Current access to HIV care includes free at point of delivery (provided by Ministry of Health clinics), as well as out-of-pocket financing and health insurance provided care at private for- and not for- profit facilities. The HIV response is funded through Ugandan Ministry of Health national budget allocations, as well as multilateral donations such as the President’s Emergency Plan for AIDS in Africa (PEPFAR) and Global Fund (GF) and other international funders. We are concerned that current funding mechanism for HIV programs in Uganda may be difficult to sustain and as service providers we are keen to explore ways in which provide lifelong HIV care to as many people living with HIV (PLHIV) as possible. Until such time as the Ugandan economy can support universal, state-supported, comprehensive healthcare, bridging alternatives must be considered. We suggest that offering patients with the sufficient means to assume some of the financial burden for their care in return for more convenient services could be one component of increasing coverage and sustaining services for those living with HIV.

## Background

Since 1990, 458 billion United States dollars (USD) has been spent in development aid for health. This aid comes primarily from donor governments and philanthropic organizations. Of this, 23.2 % was targeted for HIV/AIDs treatment and support [1]. Whilst multi-lateral funding for HIV/AIDS dramatically increased from 2004 to 2008, it has largely plateaued in the last 8 years, with a drop in 2010 and marginal 1 % increase from 2013 to 14 [2]. Across sub-Saharan Africa, up to 20 % of total spending on health is used for HIV services, and of this over 85 % is estimated to come from international funding rather than in-country sources [3]. Uganda is among 51 countries that rely on international sources to fund over 75 % of their HIV response; over 20 donors contribute over USD 400 million annually [4]. There were an estimated 1,500,000 people living with HIV in Uganda in 2012 and by the end of June 2013 567,000 eligible persons were estimated to be receiving ART. Unfortunately despite the scale up of HIV services in Uganda, there is an increased HIV prevalence (from 6.8 % in 2001 to 7.2 % in 2012) and only 40 % of people eligible for ART are currently receiving it [5]. Even at this level of ART coverage the fiscal liability for Uganda to maintain services for all those who are currently receiving it is estimated to be as much as 3 % of GDP [5, 6]. In order to meet the growing need of increased patient numbers and further ART coverage the projected costs of comprehensive HIV care and treatment services will increase substantially. Whilst this has been deemed to be cost effective in modeling in sub-Saharan African epidemics (Zambia, South Africa) expansion of ART coverage to those with CD4 counts over 350 cells/ul will still cost between $237 to $1691 per DALY averted [7]. Consequently the sustainability of these services is extremely uncertain, as international funding has plateaued and there is no clear long term plan for the Government of Uganda (GOU) to fill the potential gap. This is compounded by the growing view that HIV/AIDS care gets a disproportionate allocation of national health resources relative to other chronic conditions and that it should be “normalized” so that the financing model for HIV/AIDs more closely reflects the economic and social reality of Uganda. This article reviews the current literature on sources of HIV service financing in Uganda and the possible options to expand and sustain life-long HIV treatment in Uganda.

## Models of access to health care and HIV services in Uganda

Table 1 summarizes the ongoing local financing models in Uganda. It describes the current status of these options mentioned above, and the action required from stakeholders to expand and strengthen these options.

**Table 1:** Future HIV financing options for PNFPs

Option	Current status	Government action required	PNFP action required
Direct Government funding of services and/or centralized HIV/AIDS Fund [2, 6, 10]	Inadequate resources and inadequate budget allocation	• Streamlined, transparent public- private partnership (PPP) frameworks for health • Long term budget commitment • Engagement of donors for medium term budget support • Innovative tax/revenue collection and allocation measures • Stronger accountability and performance/quality measurement systems	• Systems to meet minimum public private partnership guidelines • Less vertical /“silo” based programs • Health system wide capacity building • Quality Assurance and Performance measurement systems
National Health Insurance Scheme [6, 11, 13]	In discussion	• Stakeholder involvement; generating a consensus on nature and scope of coverage • Fund management capacity • Governance, accountability and confidence building • Legislation and regulation	• Definition and implementation of systems to meet minimum NHIS requirements for PNFP providers • Capacity building for NHIS compliance • Quality Assurance and Performance measurement systems
Out-of-Pocket Service	Currently funds 50 % of health care [32]	• Stronger accountability and performance/quality measurement systems • Direct cash transfers	• Quality Assurance and Performance measurement systems
Private Insurance [16, 17]	Very low coverage, inadequate regulation	• Stronger Regulation • Stronger accountability and performance/quality measurement systems	• Creating an attractive business proposition for private insurance firms • Creating systems to meet minimum private health insurance company requirements
Community Health Insurance Schemes [11]	Very low coverage, inadequate regulation	• Legislation and regulation	• Community engagement and confidence building • Community accountability mechanisms • Differentiated care for scheme members
Co-Payment to subsidize overall costs of care	Used mainly by faith based organizations and NGOs, but little documentation on coverage and best practices	• No additional legislation and/or regulation	• Creating capacity to manage and report separate (paying and non-paying client) income streams • Mobilizing paying and non-paying client support • Mobilizing external stakeholder (eg donor and government) support • Maintenance of standard quality care for all clients

### Free at point of delivery

Following the abolition of user fees in Uganda in 2001 [8], health care at government clinics is free at the point of delivery, for a menu of selected services (mainly out-patient) and informal charging for additional add on services which are sometimes present. This could be termed a “publically provided health insurance” financed by taxes and other sources of government revenue. The Ministry of Health aim is to provide all necessary HIV/AIDS services free in the 2655 government health facilities it owns and manages [9]. However, the total health allocation in the national budget fell from 8.3 % in FY 2011/2012 to 7.4 % in FY 2012/2013 which means that the overall level of expenditure on health is significantly below the Abuja target of a 15 % allocation of a government’s budget to health [10], and finding sufficient funding for HIV and other health services is a challenge.

The GOU has taken some steps to introduce a National Health Insurance Scheme (NHIS) since 1995, but this has made little progress largely due to negative stakeholder influence [11]. Even if it was implemented, it’s not clear how the NHIS in its current proposed form would incorporate existing health financing systems in order to avoid fragmentation and create mechanisms for cross-subsidization [12]. Moreover, feasibility studies generally point to public mistrust, as well as a lack of key systems and capacities (particularly relating to governance and accountability) to implement the NHIS scheme in Uganda which would need to be addressed [13].

### Out-of-pocket financing

In addition to government clinic there are 994 private for profit (PFP) and 801 private not for profit (PNFP) (including faith based) health facilities, of varying quality, size and geographical distribution [9]. The 2010 National Health Accounts (NHA) Study in Uganda for health expenditures estimated that 50 % of health expenditure came from private funds (42 % from households) [14]. Many people seek HIV services with a fee for service in PFP and PNFP facilities and factors associated with this include distance to service provided, perceived better quality of service and avoidance of long waiting times [15].

### Health Insurance

Data on health insurance is challenging to come by. In 2004 WHO estimated 0.2 % of total health expenditure was on private medical insurance [16]. Estimates from 2008 suggest that only 0.5 % of the population in Uganda has any health insurance coverage [12]. Private health insurance schemes cover only 0.2 % of the population and are largely available only for people in formal employment whose employers subsidize the cost [17]. The 30 registered community insurance schemes in Uganda are mostly health facility-based schemes in which premiums are paid by individual community members. These schemes cover only an estimated additional 100,000 people (about 0.3 % of the current population) [8].

## Current Sources of funding and future options for HIV/AIDs services in Uganda

### Government funding

In FY 2012/2013 the GOU spent 7.4 % of its total annual budget on financing health systems, in particular human resources in the health sector, on which the delivery of HIV/AIDs services relies heavily. Of this only about 3 % of the health budget is for targeted to HIV/AIDS programmes, but the remainder of this funding indirectly supports HIV programmes through government health systems strengthening. Policy innovations to increase the budget for health (and specifically for HIV services) are being considered such as an HIV tax on selected services and/or a cash transfers.

### The President’s Emergency Plan for AIDS in Africa (PEPFAR) funding

The United States government Presidents Emergency Fund for AIDS Relief (PEPFAR) funded programs invested over $1.8 billion in HIV-related financing for Uganda between 2004 and 2011. PEPFAR currently contributes over 85 % of the national HIV response through projects implemented by international and local organizations (PNFP organizations) which provide free HIV/AIDS services or significantly support such services at public facilities. In practice, this means PNFPs housed within government health facilities, but running a siloed service, with directly employed health care workers but reporting to the national Ministry of Health system through the Health Information Management Service (HMIS). PEPFAR funding has already plateaued and PEPFAR now requires “cost-sharing assurances” from governments [18]. As governments assume more cost-sharing commitments, PEPFAR plans to more actively cede control over priority-setting and accounting for results to partner countries.

### Global Fund (GF) and other funders

Since its inception, the Global Fund to Fight AIDS, Tuberculosis and Malaria (Global Fund) has disbursed over USD 230 million to support various aspects of HIV/AIDS programs in Uganda through government and civil society mechanisms [19]. While the Global Fund has mainly utilized the mainstream government channels for delivering its support for the HIV effort, a substantial part of downstream resources that it provides (particularly ARVs) are delivered either directly through PNFPs or with significant support from PNFPs at government facilities [20]. The Global Fund has prioritized lower income, high burden countries and streamlined the funding application process through its new funding mechanism to make funding more predictable and flexible [21]. Nonetheless, developed country contributions to the Global Fund and other funding mechanisms are on a downward trend, triggering similar cutbacks in funding to many countries’ HIV programs. This signals potential exposure to the risk of a serious financing gap particularly for PNFPs providing free HIV services to the public.

## The case for voluntary co-pay: Can Ugandans help each other to pay for HIV care?

A National Health Insurance scheme that integrates all available external, private and public providers and financing agencies would create the opportunity reduce administrative costs, create less fragmented, full service provision points, and provide the impetus for introduction of comprehensive cross-subsidized contributory financing for health in general and HIV services in particular. However, in reality this may not be developed in time to fill the gap between drop in multi-lateral donations and a definite government commitment to funding for HIV services. As PNFP we are front line providers of HIV care. As such our organization as well as others will feel the early effects of a reduction or plateauing in funding, and will be unable to expand our services further, or will need to cut back. We believe that the option that may be most expedient for PNFP providers such as ourselves to continue to provide care in the short term is development of voluntary co-pay services. Uganda has an improving economy with a rising middle class; the number of Ugandans in the middle class (defined as those whose ability to meet their basic needs is relatively stable and who can afford to save for the future) increased from1.8 million 1992 to 12.6 million in 2012 (10 to 37 % of the population) [22]. In Uganda the 2011 AIDS indicator survey found that HIV prevalence increases with higher socio-economic status [23], and many of these PLHIV will access care in the private sector due to reduced waiting times and longer opening hours. However, the private health sector which already charges a fee for service, is a profit making sector, which has no obligation to help the lower income strata. In addition, as they are independent of the government system there is no quality control on their care [24], records are often absent and retention in care is poor [25].

We suggest that as a short term measure to mitigate against the emerging risk of financing gaps, PNFPs could explore the introduction of voluntary co-pay schemes so that those who can afford pay for services that can help subsidize the cost for those who cannot afford to pay. However, previous experience with co-pay systems both within the general health service suggest that they may affect utilization of services, as in Uganda, their abolition in 2001 increase attendance at health clinics [26]. Of particular concern in HIV care is lack of adherence to ART drugs and development of drug resistance, which has been noted when patients are paying for care [27, 28].

Therefore, in order mitigate against these issues, and to maintain a reasonable level of equity, we propose that such schemes could differentiate services through convenience and accessibility, whilst maintaining essential services at an acceptable standard for both groups of clients. For example, patients pay a standard cost for consultation in order to see the same doctor at each appointment or to access care at weekends or evenings, but are not charged for tests or medication. At the Infectious Diseases Institute in Kampala we are currently studying the feasibility of this concept by piloting a co-pay component to our large, multidisciplinary HIV clinic. A routine customer care survey of 387 of our 8500 patients suggested that over 40 % would be willing to pay a fee for greater convenience with longer (after work) opening hours and shorter waiting times. Qualitative research in our clinic has shown general acceptability of some patients for co-pay services for convenience, and also a willingness of those patients to contribute to the cost of care for other patients with less resources [29] and preliminary data suggests that access to a co-pay clinic improves retention to care in those who were lost to follow up within the private sector [30]. However, we will need to determine if this model is actually cost saving and can contribute funds to support sustaining of services, as previous experience with user fees in Africa suggest administration costs can be greater than fees collected [31].

## Conclusion

We are concerned that current funding mechanism for HIV programs in Uganda is not sustainable and as service providers we are keen to explore ways in which provide lifelong HIV care to as many people living with HIV (PLHIV) as possible. In reality, it is likely that only a model that combines several different financing approaches will provide the necessary care and treatment for our PLHIV in the future. Until such time as the Ugandan economy can support universal, state-supported, comprehensive healthcare bridging alternatives must be considered. We suggest that offering patients with the sufficient means to assume some of the financial burden for their care in return for more convenient services could be one component of preparing for a “Post-PEPFAR” funding environment. We urge other care providers to share their ideas and experiences in sustaining services so that we can learn from best practices of others in maintaining and expanding quality HIV services in resource limited settings.
